# Editorial: Molecular and Cellular Interactions Between the Host and Herpesviruses

**DOI:** 10.3389/fcimb.2021.771331

**Published:** 2021-09-23

**Authors:** Hem Chandra Jha, Subhash C. Verma

**Affiliations:** ^1^ Infection Bioengineering Group, Department of Biosciences and Biomedical Engineering, Indian Institute of Technology Indore, Indore, India; ^2^ Department of Microbiology and Immunology, University of Nevada, Reno, NV, United States

**Keywords:** herpesvirus, epstein - barr virus, kaposi sarcoma associated herpesvirus, herpes simplex virus, viral pathogenesis

Herpesviruses are among the most widely prevalent viruses in humans and associate with multiple diseases including cancer. These viruses can cause different diseases because of their propensity to infect a variety of cell types and establish latent (chronic) infection. Herpesviruses use multiple host’s cell receptors to enter the target, which determines their cellular tropism. Interestingly, virus entry and the establishment of latent infection orchestrates through various host’s and pathogen’s intricate mechanism. One such well-known mechanism is the DNA damage responses, which is efficiently manipulated by the herpesviral proteins for the establishment of viral infection and the induction of viral pathogenesis. Importantly, cells infected with herpesvirus displays two types of viral genes expression profiles, latent and lytic. The latent phase is known to be associated with the expression of minimal viral genes which are associated with maintaining viral genome and the modulation of cellular pathways. During the lytic phase, majority of the viral genes are expressed, leading to the production of progeny virions and the lysis of the infected cells. The quiescent (latent) phase, which is the predominant phase in immune competent individuals, enters into the lytic phase due to multiple cellular/environmental triggers including favorable microenvironments, immunosuppression and other associated co-infection, etc. Evidently virus infection leads to disease pathogenesis by modulating various cellular pathways, including the host cell energy metabolism pathways, for their advantage. Importantly, energy metabolism pathways such as Carbon are regulated differentially in different cells based on the cellular microenvironments. Therefore, targeting these pathways for eradicating virus associated pathogenesis is an important explorative area. Hence therapeutic agents capable of blocking host proteins, gene expression, multiple transcription factors, cellular signal pathways, immune cell activation, transcription factors, cytokines, angiogenesis, invasion, and metastasis, can be effective in preventing pathogenesis.

In this Research Topic Madavaraju et al. summarized how Herpes simplex virus (HSV) can infect a broad range of hosts that leads to human diseases. They demonstrate the importance of surface glycoproteins of HSV, which are evolutionarily conserved and show an astonishing capability to bind more than one receptor on the host cell surface. They also highlighted the importance of cellular pH in viral entry and subsequent modulation inside the host.

Next, Ohsaki and Ueda reviewed the importance of DDR activities during virus infection. They explained how virus utilizes this mechanism to make conducive microenvironments for its advantages. Further Munz has demonstrated the roles of lytic and latent genes in cancer progression. He showed the importance of lytic genes capable of making a favorable microenvironment for tumorigenesis.

Further Liu et al. reported the importance of energy metabolism in cancer progression, which included aerobic glycolysis, glutaminolysis, and fatty acid synthesis pathways. They connected the metabolism pathways with viral replication, viral survival, and proliferation in the host.

Hutcheson et al. emphasized on common cellular mutations in EBV mediated Burkitt’s lymphoma tumors and discussed how those can be targeted for the expression of viral genes that are no longer expressed, which may help in improving the treatment of EBV mediated BLs.

Bhowmik and Zhu discussed strategies utilized by herpesviruses for evading the DNA-induced immune responses, which are important in the development of vaccines and antivirals for herpesvirus-associated diseases.

Journo et al. demonstrated the footprints of cellular CpG DNA methylation in Kaposi Sarcoma tissues, which revealed a dramatic change in global methylation patterns in KS development. Further, Pardamean and Wu emphasized the studies on both SOX and ORF10 in elucidating their roles in KSHV induced disease. Authors also discussed the relatedness of these proteins in rodent virus, murine gammaherpesvirus-68 (MHV-68), and their importance in understanding KSHV pathogenesis.

Li et al. presented the importance of gene UL26.5 in Herpes simplex virus type 2. They demonstrated the immunodominance of ICP35, which can be used for designing an integrated antigen with other viral glycoproteins. Additionally, Rodriguez showed how different Sp100 isoforms are manipulated during herpesviruses HSV1, VZV, HCMV, EBV, and KSHV infection.

Dollery et al. used chronically infected derivative of TIME (telomerase immortalized endothelial) cells, which maintains latent virus by constitutively expressing (eGFP). Identity of the iTIME.219 cells was validated both phenotypically and genetically, and thus proposed to be used as a cell culture model of the KSHV replication. Further, Aalam and Totonchy evaluated KSHV biology in the lymphocyte compartment, and presented mechanisms unique to B lymphocytes.

Gou et al. investigated the metabolic networks in PK-15 cells infected with a variant virulent or classical attenuated pseudorabies virus (PRV). Authors showed that an inhibition of the pentose phosphate pathway (PPP) leads to a decline in viral titers, however the prevention of oxidative phosphorylation in the tricarboxylic acid (TCA) cycle had a minimal effect.

Barrett et al. discussed how KSHV manipulates the activities of IL-1 signaling pathway to facilitate its associated disease progression. Authors also discussed therapeutic potential of IL-1 blockade against KSHV-related diseases and several unsolved questions of the field.

Overall, this Research Topic covered viral entry, propagation inside various cells, signaling mechanism and potential therapeutics for blocking the herpesviruses associated malignancies.

## Author Contributions

The authors contributed equally to this work. HJ and SV co-wrote the manuscript. All authors contributed to the article and approved the submitted version.

## Funding

This work was supported by Ramanujan fellowship grant no. SB/S2/RIN-132/20/5 and DST-EMR: EMR/2017/001637.

## Conflict of Interest

The authors declare that the research was conducted in the absence of any commercial or financial relationships that could be construed as a potential conflict of interest.

## Publisher’s Note

All claims expressed in this article are solely those of the authors and do not necessarily represent those of their affiliated organizations, or those of the publisher, the editors and the reviewers. Any product that may be evaluated in this article, or claim that may be made by its manufacturer, is not guaranteed or endorsed by the publisher.

